# Dr. E. J. Whittleton

**Published:** 1916-12

**Authors:** 


**Dr. E. J. Whittleton,** Cleveland Homoeopathic 1884, died suddenly at his home in Webster, N. Y., Nov. 20. He was born in Walworth, June 1, 1859. He practiced in Sodus, moving to Webster about 20 years ago.

